# Dental Caries and Smoking Behaviors Among High School Students in Madinah, Saudi Arabia: A Cross-Sectional Study

**DOI:** 10.7759/cureus.77249

**Published:** 2025-01-10

**Authors:** Faisal F Hakeem, Ahmed A Marghalani, Ayah M Rashwan, Alanoud N Almohammdi, Raghad M Aljohani, Farah W Mohabat, Lubna M Helly, Hind A Almubarak, Zaina E Kalthoum

**Affiliations:** 1 Preventive Dental Sciences, College of Dentistry, Taibah University, Madinah, SAU; 2 College of Dentistry, Taibah University, Madinah, SAU

**Keywords:** adolescents, caries, oral health, saudi arabia, smoking

## Abstract

Background: Adolescence is critical for developing lifelong health habits, including oral hygiene. While the effects of smoking on oral health are well-documented in adults, research focusing on adolescents remains limited.

Objective: This paper aims to investigate the prevalence of smoking and its relationship with oral health outcomes, socioeconomic variables, and oral hygiene practices among high school students in Madinah.

Methodology: A cross-sectional study was conducted with a sample size of 2,514 high school students (*n* = 1,249, 49.7% males; *n* = 1,265, 50.3% females) from 24 schools in Madinah, Saudi Arabia. Participants completed a self-administered questionnaire that collected data on sociodemographic factors, smoking habits, and oral health behaviors. Clinical dental examinations were conducted to assess caries prevalence using the Decayed, Missing, Filled Teeth (DMFT) index. Bivariate analysis and logistic regression were used to examine the associations between smoking status and the study variables.

Results: The prevalence of smoking among participants was 8.6% (*n* = 216), with a higher proportion of males (*n* = 132 out of 1,249, 10.6%) than females (*n* = 84 out of 1,265, 6.6%) reporting tobacco use (*P* < 0.001). Smokers were more likely to attend public schools compared to nonsmokers (*n* = 190 out of 216, 88.0%, vs. *n* = 1,899 out of 2,298, 82.6%; *P* = 0.04) and had mothers with intermediate education levels (*n* = 108 out of 216, 50.0%, vs. *n* = 892 out of 2,298, 38.8%; *P* = 0.005). No significant differences were observed for the father’s education or age. Regarding oral health outcomes, smokers reported significantly poorer self-rated oral health (*n* = 43, 19.9%, vs. *n* = 328, 14.3%; *P* = 0.026), but no significant association was found between smoking status and DMFT scores (*P* = 0.66). Smokers also reported less frequent toothbrushing (*n* = 132, 61.1%, brushing regularly vs. *n* = 1,686, 73.4%, for nonsmokers; *P* = 0.001).

Conclusions: Smoking prevalence among high school students in Madinah was 8.6%, with higher rates observed among males and students attending public schools. Smoking was associated with poorer self-rated oral health and less frequent brushing habits. These findings highlight the need for targeted oral health education and smoking prevention programs in schools to address modifiable risk factors and promote healthier behaviors.

## Introduction

Smoking is a major public health concern, contributing to numerous systemic conditions worldwide [1]. Approximately 1.3 billion people use tobacco globally, making it one of the leading causes of preventable diseases [2]. Studies indicate that tobacco exposure is significantly associated with increased morbidity and mortality among children and adolescents [1]. Moreover, smoking is a well-established risk factor for oral diseases, including periodontal disease, dental caries, and oral mucosal lesions [3]. Poor oral health status can negatively impact an individual's quality of life and overall systemic health [4].

Although the association between tobacco smoking and oral health is well-documented in adults [3], research focusing on adolescents remains limited. Adolescence represents a critical period when smoking behaviors are often initiated and established [5]. In Saudi Arabia, smoking prevalence is considerable. According to the World Health Organization (WHO), 23.7% of adults and 21.2% of male adolescents reported smoking, while 9.1% of female adolescents engaged in tobacco use in 2016 [6]. Previous studies conducted in Saudi Arabia have demonstrated associations between smoking and poor oral health outcomes, including higher rates of periodontal disease, caries, and tooth discoloration [7,8]. These findings highlight the importance of addressing smoking behaviors among adolescents, as this age group is particularly vulnerable to developing long-term habits.

Adolescents are not only susceptible to initiating smoking but also face challenges in maintaining good oral health due to limited awareness and inconsistent oral hygiene practices. Smoking behavior is influenced by multiple factors, including low socioeconomic status, genetic predisposition, and social influences such as having peers or parents who smoke [9]. Initiating smoking at an early age is often associated with regular smoking in adulthood [10]. This highlights the urgent need to target smoking prevention during adolescence to reduce both short- and long-term health risks [1]. Despite these risks, tobacco use remains prevalent among adolescents in Madinah, Saudi Arabia [11]. Given the long-term implications of smoking on both oral and systemic health, the findings of this study can inform public health policies and targeted interventions aimed at reducing tobacco use and improving oral health outcomes. This study aims to assess the prevalence of smoking and its association with dental caries, socioeconomic factors, and oral hygiene behaviors among high school students in Madinah, Saudi Arabia.

## Materials and methods

Ethical approval

This study received ethical approval from Taibah University, College of Dentistry Research Ethical Committee (TUCDREC/280124). Permission was obtained from school authorities before conducting the study. Additionally, informed consent forms were collected from parents or legal guardians, before the administration of the questionnaire and clinical examination, following the guidelines of the Declaration of Helsinki.

Study design and sample selection

This study employed a cross-sectional design using a stratified multistage cluster random sampling method. Initially, a comprehensive list of high schools in Madinah city was obtained. The sampling process followed the Ministry of Education's administrative divisions, which consist of four regions: northern, eastern, western, and southern. Schools were randomly selected from each of these regions, and within each selected school, random classes were chosen to participate. Both government and private schools were included in the sampling frame. The randomization process ensured a nearly equal selection of male and female students, as well as a representative distribution across different socioeconomic groups.

Sample size calculation

The sample size was calculated using data from the latest report by the Saudi Authority of Statistics, which provided the population of male and female high school students in Madinah as 23,719 and 26,169, respectively. A 95% confidence interval (CI) and a 5% margin of error were applied, with a caries prevalence of 65%, as reported in a recent meta-analysis. Using these parameters and adjusting for the finite population of students, the required sample size for each group was determined to be 345 male students and 345 female students, resulting in a total sample size of 690. A larger sample size than the minimum requirement was collected to enhance precision and statistical power, allowing for robust subgroup analyses by gender, socioeconomic status, and parental education levels. This also accounted for potential nonresponses or exclusions due to medical or cognitive conditions.

Study population

The target population for this study was high school students in Madinah, Saudi Arabia. Inclusion criteria required students to be enrolled and attend school during the examination. Exclusion criteria included students with health conditions preventing them from understanding or completing the questionnaire and those whose parents did not consent. The selection process primarily focused on first-year high school students, as this age group has all permanent teeth (excluding third molars) erupted, making them ideal for caries assessment. However, second- and third-year students were included to enhance sample size and allow subgroup analyses. Data collection for this study was conducted between January 2023 and March 2024.

Data collection

Data were collected using a structured, self-administered questionnaire and clinical oral examinations. The questionnaire was adapted and translated from the WHO Oral Health Surveys manual [12] and collected information on demographic characteristics, socioeconomic status, oral health behaviors, and tobacco use.

Questionnaire

The questionnaire included demographic details such as sex, age, education level, and residence. Socioeconomic indicators included parental education and occupation. Participants were asked to rate their oral health as excellent, very good, good, fair, or poor and report the frequency of dental visits and toothbrushing habits. Tobacco use was assessed for various forms (cigarettes, hookah, electronic cigarettes, electronic shisha, Shamma, and tombac) with frequency options ranging from never to daily use.

Clinical examination

Examiners were trained over two days to standardize caries assessment and ensure calibration. Clinical examinations were conducted by 10 trained and calibrated dental interns to assess teeth status and calculate Decayed, Missing, Filled Teeth (DMFT) scores, using a standardized coding system developed by the WHO. The DMFT index ranges from 0 to 28, with higher scores indicating greater caries experience. Decayed teeth (D) represent untreated caries, missing teeth (M) indicate teeth lost due to caries, and filled teeth (F) represent teeth restored following caries. The total DMFT score reflects cumulative oral health conditions, with higher values suggesting poorer oral health. Examinations were conducted in schools using portable chairs, a headlight, and disposable tongue depressors. Infection control protocols were strictly maintained.

Care Index

The Care Index was calculated as the proportion of filled teeth relative to the total DMFT score, using the formula: (Filled Teeth / DMFT) × 100. This index measures the extent of restorative dental care received by participants, reflecting treatment levels.

Statistical analysis

Data analysis was conducted using Stata 18 software. The primary outcome variable, smoking behavior, was analyzed in relation to demographic, socioeconomic, and oral health factors. Participants were divided into two groups: nonsmokers and tobacco users. Descriptive statistics summarized characteristics, and bivariate associations were assessed using chi-square tests for categorical variables and t-tests for continuous variables (*P* < 0.05). Multivariate logistic regression was performed to evaluate associations between smoking and potential risk factors, including sex, age, school type, parental education, and oral hygiene behaviors. Odds ratios (ORs) with 95% CIs were reported to determine the strength of associations.

## Results

A total of 2,514 high school students participated in the study, with 1,249 males (49.7%) and 1,265 females (50.3%). The mean age of participants was 16.4 years (standard deviation [SD] = 1.0), ranging from 15 to 19 years. Smoking prevalence among the participants was 216 (8.6%), with males showing a significantly higher prevalence of smoking compared to females (61.1% vs. 38.9%, *P* < 0.001). There was no significant difference in smoking status by age (*P* = 0.06) or year of study (*P* = 0.29). Participants attending public schools had a significantly higher prevalence of smoking compared to those in private schools (8.6% vs. 6.1%, *P* = 0.04). Regarding maternal education, the tobacco users' group had a higher proportion of mothers with intermediate education levels compared to nonsmokers (50% vs. 39%, *P* = 0.005). No significant associations were observed for fathers’ education (*P* = 0.2) (Table 1).

**Table 1:** Comparison of demographic and socioeconomic factors by smoking status among study participants (n = 2,514). The chi-square test was used for categorical variables, and the t-test was used for continuous variables.

Variable	Group	Nonsmokers (*n* = 2,298, 91.4%)	Any tobacco users (*n *= 216, 8.6%)	Total (*n* = 2514)	*P*-value
Sex	Male	1,117 (48.6)	132 (61.1)	1,249 (49.7)	<0.001
	Female	1,181 (51.4)	84 (38.9)	1,265 (50.3)	
Age, years (mean ± SD)		16.4 ± 1	16.5 ± 1.2	16.41	0.06
Year of study	First year (Grade 10)	1,351 (58.8)	132 (61.1)	1,483 (59)	0.29
	Second year (Grade 11)	570 (24.8)	41 (19)	611 (24.3)	
	Third year (Grade 12)	377 (16.4)	43 (19.9)	420 (16.7)	
Mother's education	Low	345 (15.1)	30 (13.9)	375 (15)	0.005
	Intermediate	892 (39)	108 (50)	1,000 (39.9)	
	High	1,051 (45.9)	78 (36.1)	1,129 (45.1)	
Father's education	Low	216 (9.4)	25 (11.6)	241 (9.6)	0.2
	Intermediate	956 (41.8)	99 (45.8)	1,055 (42.2)	
	High	1,114 (48.7)	92 (42.6)	1,206 (48.2)	
School type	Public	1,899 (82.6)	190 (88)	2,089 (83.1)	0.04
	Private	399 (17.4)	26 (12)	425 (16.9)	

**Figure 1:**
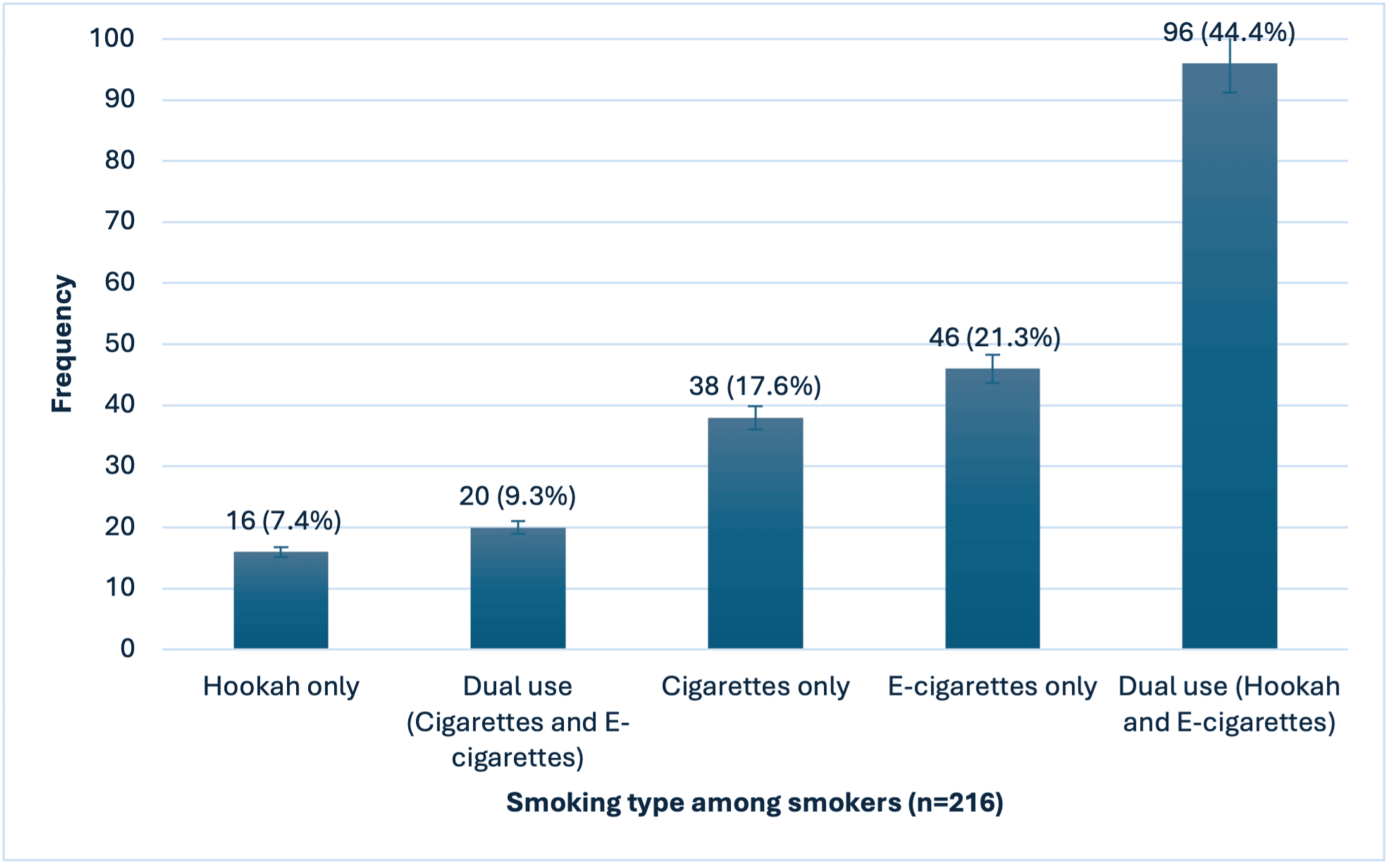
Frequency distribution of tobacco use categories among study participants (n = 216).

As shown in Figure 1, the most common smoking type among participants was dual use of hookah and e-cigarettes, reported by 96 smokers (44.4%). E-cigarettes were used only by 46 smokers (21.3%), and cigarettes were used only by 38 (17.6%). The least common types were dual use of cigarettes and e-cigarettes, reported by 20 smokers (9.3%), and hookah-only use, reported by 16 smokers (7.4%)

In terms of oral health status, no significant differences were observed between smokers and nonsmokers for DMFT scores (mean 3.7 for smokers vs. 4.1 for nonsmokers, *P* = 0.66), decayed teeth (D), or filled teeth (F) scores (Table 2). However, a slight but statistically significant difference was found in missing teeth (M), with smokers having fewer missing teeth than nonsmokers (*P* = 0.04). The Care Index did not differ significantly between the groups (*P* = 0.76). Self-rated oral health was significantly associated with smoking status, with 43 (19.9%) smokers rating their oral health as poor or fair compared to 328 (14.3%) nonsmokers (*P* = 0.026). Additionally, brushing frequency was significantly different between smokers and nonsmokers (*P* = 0.001), with a higher proportion of smokers reporting never brushing their teeth (6.9% vs. 3.6%) or brushing rarely (19.4% vs. 13.1%).

**Table 2:** Summary of oral health status, dental visits, and brushing habits by smoking status for study participants (n = 2,514). The chi-square test was used for categorical variables, and the t-test was used for continuous variables.

Variable	Categories	Nonsmokers (*n *= 2,298, 91.4%)	Any tobacco users (*n *= 216, 8.6%)	Total (*n *= 2,514)	*P*-value
D (mean ± SD)		3.4 ± 3.1	3.2 ± 3.0	3.4 ± 3.1	0.98
M (mean ± SD)		0.5 ± 1.2	0.4 ± 0.9	0.5 ± 1.2	0.04
F (mean ± SD)		0.2 ± 0.5	0.2 ± 0.5	0.2 ± 0.5	0.64
DMFT (mean ± SD)		4.1 ± 3.7	3.7 ± 3.4	4.1 ± 3.6	0.66
Care Index (%)		3.6%	4.1%	3.6%	0.76
Self-rated oral health	Good/Very good/Excellent	1,967 (85.7)	173 (80.1)	2,140 (85.2)	0.026
	Fair/poor	328 (14.3)	43 (19.9)	371 (14.8)	
Dental visits frequency during last year	Never received dental care	450 (19.7)	49 (22.7)	499 (19.9)	0.161
	Once	280 (12.2)	16 (7.4)	296 (11.8)	
	Twice	384 (16.8)	33 (15.3)	417 (16.6)	
	Thrice or more	675 (29.5)	62 (28.7)	737 (29.4)	
	Did not visit during last year	501 (21.9)	56 (25.9)	557 (22.2)	
Brushing frequency	Never	82 (3.6)	15 (6.9)	97 (3.9)	0.001
	Rare	300 (13.1)	42 (19.4)	342 (13.6)	
	Irregular brushing	227 (9.9)	27 (12.5)	254 (10.1)	
	Regular brushing	1,682 (73.4)	132 (61.1)	1,814 (72.4)	

Logistic regression analysis indicated that females had significantly lower odds of smoking compared to males (OR = 0.63, 95% CI 0.47-1.10; p = 0.005; Table 3). School type was marginally associated with smoking, with students in private schools having lower odds of smoking compared to those in public schools (OR = 0.65, 95% CI 0.41-1.03; *P* = 0.069). Maternal education showed a significant association with smoking, with students whose mothers had intermediate education having higher odds of smoking compared to those with low maternal education (OR = 1.62, 95% CI 1.05-2.50; *P* = 0.029). Regular brushing was associated with lower odds of smoking (OR = 0.54, 95% CI 0.29-1.00; *P* = 0.053), while self-rated oral health was not significantly associated with smoking status (*P* = 0.15). Age was also marginally associated with smoking (*P* = 0.058), suggesting that older students may be more likely to smoke.

**Table 3:** Logistic regression showing the association between smoking status and potential risk factors among high school students in Madinah, Saudi Arabia (n = 2,514). *P < 0.1. **P < 0.01. ***P < 0.05. OR, odds ratios

	Smoking	OR	95% confidence interval	*P*-value	Sig
Age (Years)		1.14	0.95-1.37	0.058	*
Sex	Male	Reference			
	Female	0.63	0.47-1.10	0.005	**
School type	Governmental	Reference			
	Private	0.65	0.41-1.03	0.069	*
Mother's education	Low	Reference			
	Medium	1.62	1.05-2.50	0.029	***
	High	1.17	0.74-1.87	0.486	
Brushing frequency	Never	Reference			
	Rare	0.75	0.39-1.43	0.39	
	Irregular	0.70	0.35-1.41	0.32	
	Regular	0.54	0.29-1.00	0.053	*
Self-rated oral health	Good/Very good/Excellent	Reference			
	Fair/poor	1.30	0.90-1.87	0.15	

## Discussion

This study assessed the prevalence of smoking and its association with oral health outcomes, sociodemographic factors, and oral hygiene behaviors among high school students in Madinah, Saudi Arabia. The findings highlight several key trends and associations that contribute to our understanding of adolescent smoking behavior and oral health outcomes.

The prevalence of smoking in this study was 8.6%, which is lower than previous studies conducted in Madinah, such as the 2015 study reporting a prevalence of 15.1% [9]. This difference may reflect changes in smoking behaviors over time, differences in sample selection methods, or variations in the characteristics of the studied populations, such as age groups, gender distribution, and socioeconomic background. For example, this study included both public and private schools and utilized a larger and more diverse sample size, which may have contributed to lower reported prevalence rates. Consistent with prior research [13,14], male students had a significantly higher prevalence of smoking than females, reinforcing gender disparities in tobacco use. Cultural norms and greater social restrictions on female smoking may explain these differences, as smoking is more socially acceptable for males in Saudi society. These findings highlight the need for gender-specific interventions targeting smoking prevention and cessation.

Patterns of smoking behavior revealed that the dual use of hookah and e-cigarettes was the most prevalent, followed by the exclusive use of e-cigarettes and cigarettes. These findings align with previous studies [15,16], reflecting the growing popularity of e-cigarettes among adolescents. This trend emphasizes the need for youth-focused prevention programs to address the appeal and accessibility of e-cigarettes.

Socioeconomic factors also played a role. Students attending public schools exhibited higher smoking rates compared to those in private schools, potentially reflecting differences in economic resources, access to extracurricular activities, and school-based health programs. Maternal education emerged as a significant predictor, with intermediate maternal education levels defined as completion of secondary school with higher odds of smoking. These findings suggest that family background and education may influence adolescent smoking habits, possibly due to differences in parental awareness about the health risks of smoking or lower emphasis on smoking prevention. Addressing these disparities requires interventions such as school-based education programs, parental counseling initiatives, and community engagement activities to raise awareness and provide resources for smoking prevention and cessation.

In terms of oral health outcomes, no significant differences were observed in DMFT scores between smokers and nonsmokers, contradicting some prior studies [17,18]. However, smokers were more likely to report poorer self-rated oral health and less frequent tooth brushing. These behavioral patterns may partially explain the absence of significant DMFT differences, as smokers may neglect oral hygiene practices, leading to cumulative risks over time rather than immediate impacts detectable in cross-sectional data [19,20].

Interestingly, smokers had fewer missing teeth than nonsmokers, which may be attributed to the relatively young age range of participants (15-19 years) and the short duration of smoking exposure in this population. However, this finding warrants further investigation in longitudinal studies to assess the long-term effects of smoking on tooth retention and periodontal health. Future research should focus on monitoring changes in dental and periodontal status over time, including the progression of tooth loss, periodontal attachment loss, and the onset of oral mucosal conditions associated with smoking.

The findings emphasize the need for interventions targeting smoking prevention and cessation among adolescents, particularly focusing on e-cigarette regulation, public school environments, and parental education. Oral health education programs should be integrated into school curricula to promote proper hygiene practices and raise awareness about the risks of smoking.

Limitations of this study include reliance on self-reported data, which may introduce recall bias and social desirability bias, particularly regarding smoking behaviors, as participants may underreport smoking due to cultural and social norms. This may have contributed to the observed sex differences, with females potentially underreporting tobacco use more than males. Additionally, the cross-sectional design precludes establishing causality. Variability in examiner assessments was minimized through calibration training, but minor discrepancies cannot be ruled out. Despite these limitations, the study's strengths include its large, representative sample, standardized data collection methods, and alignment with WHO criteria, making the findings generalizable to similar populations.

Future research should consider longitudinal designs to track changes in smoking behavior and oral health outcomes over time, as well as qualitative approaches to explore the underlying motivations for tobacco use. Further studies could also examine the impact of family dynamics, peer influence, and media exposure on smoking initiation.

## Conclusions

This study highlights the association between adolescent smoking behaviors, oral health outcomes, and socioeconomic factors among high school students in Madinah, Saudi Arabia. The findings emphasize the need for targeted smoking prevention programs, regulation of emerging tobacco products, and school-based oral health education initiatives. Specific actions, such as parental counseling and community awareness campaigns, should also be prioritized to reduce smoking prevalence and promote better oral health outcomes in adolescents.
